# Radiographic accuracy of implant positioning in unicompartmental knee arthroplasty, does an imageless robotic system make a difference?

**DOI:** 10.1007/s00402-026-06499-7

**Published:** 2026-10-03

**Authors:** Alice Montagna, Luca Andriollo, Virginia Cinelli, Marina Marescalchi, Luca Matascioli, Francesco Benazzo, Stefano Marco Paolo Rossi

**Affiliations:** 1 Fondazione Poliambulanza Istituto Ospedaliero, Brescia, Italy; 2 Policlinico San Matteo Fondazione, Pavia, Italy; 3 Catholic University of the Sacred Heart, Rome, Italy; 4 Fondazione Poliambulanza Istituto Ospedaliero, Brescia, Italy; 5 Istituto Universitario di Studi Superiori di Pavia, Pavia, Italy; 6 OrtoTrauma Department, Ospedale Niguarda Ca❜ Granda, Milan, Italy; 7 Link Campus University, Rome, Italy

**Keywords:** Unicompartmental knee arthroplasty, Robotic-assisted surgery, Radiographic alignment, Implant positioning, Coronal alignment

## Abstract

**Background:**

Robotic-assisted technologies and evolving implant designs have been introduced in unicompartmental knee arthroplasty (UKA) to improve the accuracy and reproducibility of component positioning. However, the relative contribution of robotic assistance compared with conventional instrumentation to radiographic outcomes in experienced, high-volume centers remains unclear.

**Methods:**

This retrospective comparative study included 96 consecutive medial UKAs performed at a single high-volume institution. Patients were stratified into three groups: Journey I UKA implanted with NAVIO imageless robotic assistance (*n* = 38), Journey II Uni UKA implanted using conventional instrumentation (*n* = 27), and Journey II UK UKA implanted with CORI imageless robotic assistance (*n* = 31). Standardized standing long-leg weight-bearing radiographs were obtained preoperatively and postoperatively. Radiographic evaluation included hip–knee–ankle (HKA) angle, femoral component coronal angle (FCCA), tibial component coronal angle (TCCA), and sagittal alignment parameters. Tibial coronal alignment was interpreted according to the Cartier metaphyseal alignment philosophy. Intergroup comparisons were performed using appropriate statistical tests.

**Results:**

Baseline demographic characteristics and preoperative radiographic parameters were comparable among the three groups (all *p* > 0.05). Postoperative mean HKA was 177.1° ± 2.1° in the Journey I NAVIO group, 177.3° ± 2.5° in the Journey II Uni conventional group, and 177.7° ± 2.2° in the Journey II UK CORI group, with no statistically significant differences (*p* = 0.56). Femoral and tibial component coronal alignment, including TCCA, showed no significant intergroup differences. All cases across the three cohorts were within predefined safe zones for HKA, FCCA, and TCCA. Sagittal alignment parameters were also comparable between groups.

**Conclusion:**

In a high-volume center adopting a consistent alignment philosophy, medial UKA achieves equivalent and highly accurate radiographic outcomes regardless of robotic assistance or conventional instrumentation.

## Introduction

Unicompartmental knee arthroplasty (UKA) is an established surgical option for the treatment of isolated medial compartment osteoarthritis, offering advantages over total knee arthroplasty such as bone preservation, faster recovery, and maintenance of more physiological knee kinematics [1]. Nevertheless, the success of UKA is highly dependent on accurate restoration of coronal limb alignment and precise component positioning [2, 3].

To address variability associated with manual instrumentation, robotic-assisted UKA systems were introduced, providing computer-guided bone resections, real-time alignment feedback, and intraoperative assessment of limb kinematics [4, 5]. Early clinical and radiographic studies demonstrated encouraging accuracy with robotic assistance [2, 3, 6, 7] and after image based platforms also imageless systems such as NAVIO Smith & Nephew (Memphis, TN, USA) were introduced. More recently, the CORI Surgical System has been developed by the same company as an evolution of imageless robotic technology, incorporating refinements in workflow, computational power, and intraoperative feedback.

In parallel with technological developments, unicompartmental implant designs have evolved along different conceptual pathways. Importantly, contemporary UKA systems do not represent a single linear design evolution. The Journey I unicompartmental system represents an early fixed-bearing design developed specifically for UKA. The Journey II Uni system was conceived as an anatomical evolution of this concept and is implanted using conventional instrumentation. In contrast, the Journey II UK system represents a distinct implant lineage derived from the ZUK (Zimmer Uni Knee acquired by S&N) unicompartmental knee, with different geometric and biomechanical principles.

Despite these parallel developments, few studies have compared different robotic platforms and implant systems within a single institution using standardized radiographic protocols. The relative contribution of robotic assistance versus implant system to coronal radiographic accuracy therefore remains unclear.

The aim of this study was to compare radiographic coronal limb alignment and component positioning among three UKA configurations: Journey I with NAVIO robotic assistance, Journey II Uni with conventional instrumentation, and Journey II UK with CORI robotic assistance. We hypothesized that, when performed in a high-volume center using standardized indications and surgical techniques, all three configurations would achieve comparable radiographic accuracy.

## Materials and methods

### Study design and patient selection

This retrospective comparative study analyzed consecutive patients undergoing medial unicompartmental knee arthroplasty (UKA) for isolated medial compartment osteoarthritis at a single high-volume knee arthroplasty center. All procedures were performed by the same experienced surgical team following uniform indications, surgical philosophy, and perioperative protocols.

Patients were stratified according to implant system and surgical technique into three cohorts:

- Group 1: Journey I UKA implanted with NAVIO imageless robotic assistance (*n* = 38) operated between January 2020 and December 2021.
- Group 2: Journey II Uni UKA implanted with conventional manual instrumentation (*n* = 27) operated between January 2018 and December 2019.
- Group 3: Journey II UK UKA implanted with CORI imageless robotic assistance (*n* = 31) operated between January 2024 and December 2025.

Cases were performed over different time periods, reflecting the progressive adoption of different implant systems and enabling technologies, while maintaining identical clinical indications and radiographic assessment protocols.

### Inclusion and exclusion criteria

Indications for medial UKA were identical across all groups and mirrored those adopted in prior long-term and navigated UKA studies from the same institution.

Inclusion criteria were:

- Symptomatic isolated medial compartment osteoarthritis.
- Kellgren–Lawrence grade ≥ 3 in the medial compartment.
- Minimum knee flexion ≥ 90°.
- Maximum extension deficit ≤ 10°.
- Correctable varus deformity ≤ 15°.
- Preserved lateral compartment joint space on weight-bearing radiographs.
- Intact cruciate ligaments.

Exclusion criteria included:

- Symptomatic patellofemoral osteoarthritis.
- Lateral compartment degeneration > Kellgren–Lawrence grade 2.
- Inflammatory arthropathies.
- Post-infectious arthritis.
- Fixed coronal or sagittal deformities not correctable intraoperatively.
- Diffuse tricompartmental osteoarthritis.

Minor chondral changes in the lateral or patellofemoral compartments were not considered contraindications. Final eligibility was systematically confirmed by **intraoperative inspection**.

### Implants and surgical technique

All implants were manufactured by Smith & Nephew (Memphis, TN, USA).

- Journey I UKA is a fixed-bearing unicompartmental system with a polyradial Oxinium femoral component and a flat polyethylene tibial insert.
- Journey II Uni UKA represents an anatomical evolution of the Journey I concept and is implanted using conventional instrumentation.
- Journey II UK UKA represents a distinct unicompartmental implant lineage derived from the original ZUK design philosophy, implanted in this series using robotic assistance.

All procedures were performed through a minimally invasive medial parapatellar approach.

### Tibial cut philosophy and Cartier

Across all groups, the tibial cut was oriented according to the Cartier alignment philosophy [2, 8–10], aiming to respect the native metaphyseal varus of the proximal tibia rather than enforcing a neutral mechanical tibial cut.

The Cartier angle is defined as the angle between:

- the line perpendicular to the tibial mechanical axis, and.
- the line perpendicular to the tangent of the lateral tibial plateau.

This concept was applied consistently in all cases, regardless of the use of robotic assistance or conventional instrumentation.

- In Groups 1 and 3, the Cartier angle target was reproduced intraoperatively using imageless robotic planning, with real-time feedback on tibial coronal orientation.
- In Group 2, the tibial cut was performed using standard extramedullary instrumentation, aiming to reproduce the same metaphyseal varus orientation according to the Cartier principle.

All components were cemented. Surgical objectives included restoration of the native joint line, avoidance of tibial overcorrection into valgus, preservation of bone stock, and achievement of balanced medial compartment kinematics throughout the range of motion. Postoperative rehabilitation followed a standardized protocol with immediate full weight-bearing.

### Radiographic evaluation

Standardized standing long-leg weight-bearing radiographs, using the same protocol over time, were obtained preoperatively and postoperatively for all patients. Radiographic analysis was conducted according to the methodology described by Khow et al. [11]. All radiographic measurements were performed using the Picture Archiving and Communication Systems (Carestream Health, Rochester, NY). Measurements were recorded to an accuracy of 0.1° The following measures were collected, and the results classified as within or without the so called “safe zone” as defined by the authors which is included within a 3 to 4 degrees margin. All the radiographic results are summarized in Tables 1, 2, 3 and 4.

**Table 1:** Patient demographics and baseline characteristics

Parameter	Journey I + NAVIO (*n* = 38)	Journey II Uni + conventional (*n* = 27)	Journey II UK + CORI (*n* = 31)	*p* value
Age (years), mean ± SD	66.8 ± 7.9	67.4 ± 8.3	66.1 ± 7.6	0.84
Female sex, *n* (%)	26 (68.4%)	18 (66.7%)	21 (67.7%)	0.98
BMI (kg/m²), mean ± SD	27.4 ± 3.8	27.1 ± 4.1	27.6 ± 3.5	0.89
Side (Right / Left)	21 / 17	15 / 12	17 / 14	0.99
Preoperative HKA (°), mean ± SD	171.8 ± 3.2	172.1 ± 3.5	171.6 ± 3.1	0.87

**Table 2:** Preoperative radiographic alignment parameters

Parameter	Journey I + NAVIO	Journey II Uni + Conventional	Journey II UK + CORI	*p* value
Hip–knee–ankle angle (°)	173.3 ± 2.9	175.4 ± 2.8	174.8 ± 3.0	> 0.05
Lateral distal femoral angle (°)	89.5 ± 2.4	88.2 ± 2.2	89.1 ± 2.3	> 0.05
Medial proximal tibial angle (°)	86.7 ± 2.4	85.9 ± 2.8	86.3 ± 2.5	> 0.05
Joint line convergence angle (°)	4.1 ± 1.3	3.5 ± 1.1	3.8 ± 1.2	> 0.05

**Table 3:** Postoperative radiographic outcomes

Parameter	Journey I + NAVIO	Journey II Uni + conventional	Journey II UK + CORI	*p* value
Postoperative HKA (°), mean ± SD	177.1 ± 2.1	177.3 ± 2.5	177.7 ± 2.2	0.56
Femoral component coronal angle (FCCA, °)	90.6 ± 2.4	91.1 ± 2.6	90.8 ± 2.3	0.71
Tibial component coronal angle (TCCA, °)	87.9 ± 2.7	88.3 ± 2.9	88.1 ± 2.5	0.83
Tibial slope (°)	4.9 ± 1.0	4.7 ± 1.2	4.8 ± 1.1	0.88
Femoral sagittal axis (°)	22.1 ± 3.1	21.8 ± 2.9	22.0 ± 3.0	0.91

**Table 4:** Safe-zone compliance

Parameter	Journey I + NAVIO (%)	Journey II Uni + conventional (%)	Journey II UK + CORI (%)	*p* value
HKA within safe zone (177° ± 3°)	100	100	100	–
FCCA within safe zone (± 5°)	100	100	100	–
TCCA within Cartier-based safe zone (± 3°)	–	–	–	–

The following parameters were evaluated:

- Hip–knee–ankle (HKA) angle.
- Femoral component coronal angle (FCCA).
- Tibial component coronal angle (TCCA).

Tibial coronal orientation was interpreted in relation to the Cartier alignment concept, distinguishing physiological metaphyseal varus positioning from excessive varus or valgus malalignment.

Two independent observers, blinded to group allocation, performed all measurements.

Safe zones were defined as:

- HKA: 177° ± 3°
- FCCA: within ± 5° from neutral.
- TCCA: within ± 3° from the intended metaphyseal orientation.

### Statistical analysis

Continuous variables were expressed as mean ± standard deviation (SD). Normality was assessed using the Shapiro–Wilk test.

Intergroup comparisons were performed using:

- One-way ANOVA for normally distributed variables.
- Kruskal–Wallis test for non-normally distributed variables.

Categorical variables were compared using the chi-square test. Statistical significance was set at *p* < 0.05.

A formal a priori power analysis was not performed due to the retrospective nature of the study. However, the present sample size is comparable to previously published radiographic UKA studies evaluating component positioning accuracy. The relatively limited size of each cohort should therefore be interpreted as a potential limitation when detecting small intergroup differences.

## Results

A total of 96 medial UKAs were included in the analysis. Baseline demographic characteristics and preoperative radiographic parameters were comparable among the three groups, with no statistically significant differences in age, sex distribution, laterality, or preoperative HKA (all *p* > 0.05). (Table 1) Preoperative coronal alignment demonstrated a varus pattern typical of isolated medial compartment osteoarthritis.

### Postoperative coronal limb alignment

All three groups showed effective correction toward physiological coronal alignment without systematic overcorrection (Tables 1 and 2).

Postoperative mean HKA values were:

- 177.1° ± 2.1° in the Journey I NAVIO group.
- 177.3° ± 2.5° in the Journey II Uni conventional group.
- 177.7° ± 2.2° in the Journey II UK CORI group.

No statistically significant intergroup differences were observed (*p* = 0.56). Safe-zone compliance for postoperative HKA reached 100%, with no significant differences in the proportion of outliers.

### Tibial component coronal alignment and Cartier angle consistency

- The TCCA (tibial component coronal angle) is the angle between a parallel line to the tibial component and a perpendicular line to the tibial mechanical axis. The TCCA, which is related to the proximal tibial varus angle (so called Cartier angle) was respected in all patients with a mean TCCA value of 2,1 ° (SD 1.3) no valgus cuts (or “out of the safe zone) were identified. No statistical difference was found in this measure between the three groups. Mean postoperative TCCA values were within the predefined target range in all three cohorts, reflecting consistent reproduction of the Cartier metaphyseal varus orientation of the proximal tibia. No statistically significant differences were detected between robotic-assisted and conventionally performed UKA in terms of tibial coronal alignment. The proportion of tibial component outliers relative to the intended metaphyseal orientation was comparable across groups.
- Concerning the lateral view measures were analyzed according to Kayabicer et al. [12]. Using the flexion angle relative to the midline sagittal the mean femoral axis was 22 degrees (SD 3) with no statistical difference between groups while the mean tibial slope was 4.8° (SD 1.1) with no statistical difference between groups. (Tables 3 and 4)

### Femoral component coronal alignment

- The FCCA (femoral component coronal angle) is the absolute angle between the femoral component long axis and femoral mechanical axis. The FCCA,. was less than 3 degrees (< 3°) cases and in 6 cases between 3 and 4° (< 4°), being in all cases within the so called “safe zone”. No statistical difference was found in this measure between the two groups. Femoral component positioning was accurate and reproducible across all three groups. Mean FCCA values were within predefined safe zones, with no statistically significant intergroup differences. (Tables 3 and 4)

### Summary of radiographic outcomes

Across all evaluated parameters, Journey I NAVIO robotic-assisted UKA, Journey II Uni conventionally implanted UKA, and Journey II UK CORI robotic-assisted UKA demonstrated equivalent radiographic accuracy in coronal limb alignment and component positioning. Importantly, consistent reproduction of the Cartier tibial alignment philosophy was achieved independently of the use of robotic assistance.

## Discussion

The most important finding of the present study is that accurate and reproducible radiographic alignment in medial unicompartmental knee arthroplasty (UKA) can be achieved independently of an imageless robotic assistance when surgery is performed in a high-volume center adopting a consistent alignment philosophy. Specifically, no statistically significant differences were observed in coronal limb alignment or component positioning among Journey I UKA implanted with NAVIO imageless robotic assistance, Journey II Uni UKA implanted using conventional instrumentation, and Journey II UK UKA implanted with CORI imageless robotic assistance.

Component malpositioning and coronal malalignment have long been implicated as major contributors to early UKA failure, and robot-assisted technologies were developed, in part, to improve precision and reduce radiographic outliers compared with conventional techniques. A meta-analysis of over 2,700 UKA procedures reported that robotic assistance was associated with improved component alignment accuracy and fewer mechanical axis outliers than conventional instrumentation, although functional outcomes did not consistently differ between groups [13].

More recent evidence has shifted attention not only to the presence of robotics but also to the type of robotic guidance used. Systematic review data indicate that image-based robotic UKA systems, which utilize preoperative CT or other advanced imaging to build a detailed three-dimensional anatomical model, are associated with fewer radiologic outliers in hip–knee–ankle axis and joint line height when compared with imageless robotic systems [14]. Similarly, cohort studies have found that medial UKAs performed with image-based robotic assistance demonstrate higher accuracy in restoring intended limb alignment and implant positioning compared with imageless robotic systemswhen assessed with rigorous radiographic criteria [15].

In contrast, imageless robotic systems offer distinct practical advantages, including avoidance of preoperative imaging and associated radiation exposure, lower logistical burden, and adaptability to intraoperative anatomical findings without the need for CT planning. These systems remain widely used in clinical practice and have demonstrated excellent radiographic outcomes in registry and cohort studies [16]. In the present series, both imageless platforms (NAVIO and CORI) delivered highly accurate coronal and sagittal alignment with minimal outliers within predefined safe zones.

A key strength of this study is the uniform adoption of the Cartier alignment philosophy for proximal tibial alignment across all groups [8–10]. By intentionally respecting the native metaphyseal varus rather than enforcing a neutral mechanical cut, consistent tibial component orientation was achieved regardless of technology. This reinforces the concept that alignment intent and surgical philosophy may outweigh the specific robotic modality in determining radiographic accuracy when expertise is high [17].

Although sagittal alignment parameters (e.g., posterior tibial slope and femoral sagittal axis) were measured and found to be equivalent among groups, the present analysis remains limited to static radiographs. Rotational alignment and dynamic soft-tissue behavior were not evaluated, and such subtleties may be better elucidated with three-dimensional imaging or advanced dynamic assessment tools.

This study has several limitations that should be acknowledged. First, its retrospective design and single-center setting may limit the generalizability of the findings. However, the use of consecutive cases, standardized indications, and uniform surgical philosophy reduces selection bias and strengthens internal validity. Second, the three study groups were treated during different time periods.The sequential adoption of implant systems and enabling technologies reflects the natural evolution of surgical practice within the institution. Although surgical indications, alignment philosophy, and radiographic protocols remained unchanged, temporal factors such as increasing surgical experience, improvements in perioperative workflow, and advances in imaging systems may represent potential confounders. Consequently, the absence of radiographic differences between groups should be interpreted with caution, as incremental surgical refinement over time cannot be completely excluded Third, the analysis was intentionally limited to static radiographic parameters obtained from standard weight-bearing long-leg radiographs. Rotational alignment, dynamic soft-tissue behavior, and in vivo kinematics were not evaluated and may reveal differences not captured by conventional radiographic assessment. Finally, clinical outcomes, patient-reported outcome measures, and implant survivorship were not analyzed. While this represents a limitation, it also reflects the focused aim of the study to isolate radiographic alignment and component positioning as a single explanatory factor. The present analysis focuses exclusively on radiographic alignment and component positioning. Radiographic measurements were performed by two independent observers to reduce observer-related bias. Although reliability testing demonstrated good agreement between observers, minor measurement variability inherent to long-leg radiographic assessment cannot be completely eliminated. Although accurate component positioning is considered an important determinant of implant longevity in unicompartmental knee arthroplasty, radiographic alignment alone does not necessarily translate directly into improved clinical outcomes or implant survivorship. Several studies have shown that comparable radiographic alignment may coexist with similar functional outcomes regardless of the surgical technology employed. Therefore, while the current findings support the reproducibility of the alignment philosophy adopted in this series, long-term clinical outcomes and survivorship analyses are necessary to determine the true clinical impact of these radiographic findings.

## Conclusions

In a high-volume center adopting a consistent Cartier-based alignment philosophy, medial unicompartmental knee arthroplasty achieves equivalent and highly accurate radiographic outcomes with imageless robotic assistance or conventional instrumentation. These findings suggest that surgical experience and alignment intent may outweigh the specific enabling technology in determining radiographic accuracy, while the clinical relevance of differences between robotic platforms requires further investigation.

## Data Availability

Data are stored in a separate data repository and available upon request.

## References

[1] Johal S, Nakano N, Baxter M et al (2018) Unicompartmental Knee Arthroplasty: The Past, Current Controversies, and Future Perspectives. J Knee Surg 31:992–998. 10.1055/s-0038-1625961 29514367

[2] Zambianchi F, Franceschi G, Rivi E et al (2019) Does component placement affect short-term clinical outcome in robotic-arm assisted unicompartmental knee arthroplasty? Bone Joint J 101-B:435–442. 10.1302/0301-620X.101B4.BJJ-2018-0753.R1 30929494

[3] Matassi F, Innocenti M, Giabbani N et al (2022) Robotic-assisted unicompartmental knee arthroplasty reduces components’ positioning differences among high- and low-volume surgeons. J Knee Surg 35:1549–1555. 10.1055/s-0041-1727115 33853154

[4] Leggieri F, Martín Cocilova FN, Civinini A et al (2025) Robotic-assisted joint line preserving unicompartmental knee arthroplasty positioning among octogenarians. J Pers Med 15:362. 10.3390/jpm15080362 PMC12387272 40863424

[5] Innocenti M, Leggieri F, Chirico M et al (2025) Comparable clinical outcomes between octogenarians and young patients following robotic-assisted unicompartmental knee arthroplasty. J Exp Orthop 12:e70491. 10.1002/jeo2.70491 PMC12560248 41164326

[6] Van Der List JP, Chawla H, Joskowicz L, Pearle AD (2016) Current state of computer navigation and robotics in unicompartmental and total knee arthroplasty: a systematic review with meta-analysis. Knee Surg Sports Traumatol Arthrosc 24:3482–3495. 10.1007/s00167-016-4305-9 27600634

[7] Batailler C, White N, Ranaldi FM et al (2019) Improved implant position and lower revision rate with robotic-assisted unicompartmental knee arthroplasty. Knee Surg Sports Traumatol Arthrosc 27:1232–1240. 10.1007/s00167-018-5081-5 30066017

[8] Cartier P, Sanouiller J-L, Grelsamer RP (1996) Unicompartmental knee arthroplasty surgery. J Arthroplasty 11:782–788. 10.1016/S0883-5403(96)80177-X 8934317

[9] Cartier P (2014) Story of My Passion. Knee 21:349–350. 10.1016/j.knee.2013.11.007 24439766

[10] Rivière C, Sivaloganathan S, Villet L et al (2022) Kinematic alignment of medial UKA is safe: a systematic review. Knee Surg Sports Traumatol Arthrosc 30:1082–1094. 10.1007/s00167-021-06462-6 33743031

[11] Khow YZ, Liow MHL, Lee M et al (2021) Coronal Alignment of Fixed-Bearing Unicompartmental Knee Arthroplasty Femoral Component May Affect Long-Term Clinical Outcomes. J Arthroplasty 36:478–487. 10.1016/j.arth.2020.07.070 32828619

[12] Kaya Bicer E, Servien E, Lustig S et al (2010) Sagittal flexion angle of the femoral component in unicompartmental knee arthroplasty: is it same for both medial and lateral UKAs? Knee Surg Sports Traumatol Arthrosc 18:928–933. 10.1007/s00167-010-1063-y 20130835

[13] Chin BZ, Tan SSH, Chua KCX et al (2021) Robot-Assisted versus Conventional Total and Unicompartmental Knee Arthroplasty: A Meta-analysis of Radiological and Functional Outcomes. J Knee Surg 34:1064–1075. 10.1055/s-0040-1701440 32185785

[14] Tomescu H, Avram GM, Pacchiarotti G et al (2025) Image-Based Robotic Unicompartmental Knee Arthroplasty Results in Fewer Radiologic Outliers with No Impact on Revision Rates Compared to Imageless Systems: A Systematic Review. J Clin Med 14:5996. 10.3390/jcm14175996 PMC12428876 40943763

[15] Gaggiotti S, Foissey C, Pineda T et al (2024) Enhancing robotic precision in medial UKA: Image-based robot‐assisted system had higher accuracy in implant positioning than imageless robot‐assisted system across 292 knees. Knee Surg Sports Traumatol Arthrosc 32:2097–2106. 10.1002/ksa.12215 38690988

[16] El Motassime A, Fulli L, Andriollo L et al (2026) Robotics total knee arthroplasty: is an image-based the only solution? Front Surg 12:1732887. 10.3389/fsurg.2025.1732887 PMC12872902 41657739

[17] Andriollo L, Montagna A, Mazzella GG et al (2024) Navigated versus conventional medial unicompartmental knee arthroplasty: minimum 18 years clinical outcomes and survivorship of the original Cartier design. Knee 49:183–191. 10.1016/j.knee.2024.07.009 39043013

